# Evaluating the accuracy of automated cephalometric analysis based on artificial intelligence

**DOI:** 10.1186/s12903-023-02881-8

**Published:** 2023-04-01

**Authors:** Han Bao, Kejia Zhang, Chenhao Yu, Hu Li, Dan Cao, Huazhong Shu, Luwei Liu, Bin Yan

**Affiliations:** 1 Department of Orthodontics, The Affiliated Stomatological Hospital of Nanjing Medical University, Nanjing, 210029 China; 2 Jiangsu Province Key Laboratory of Oral Diseases, Nanjing Medical University, Nanjing, 210029 China; 3 Jiangsu Province Engineering Research Center of Stomatological Translational Medicine, Nanjing Medical University, Nanjing, 210029 China; 4 Laboratory of Image Science and Technology, Southeast University, Nanjing, 210096 China; 5 Centre de Recherche en Information Biomédicale Sino-Français, Rennes, 35000 France; 6 Jiangsu Provincial Joint International Research Laboratory of Medical Information Processing, Southeast University, Nanjing, 210096 China

**Keywords:** Cephalometric analysis, Automatic identification, Cone-beam CT, Artificial intelligent

## Abstract

**Background:**

The purpose of this study was to evaluate the accuracy of automatic cephalometric landmark localization and measurements using cephalometric analysis via artificial intelligence (AI) compared with computer-assisted manual analysis.

**Methods:**

Reconstructed lateral cephalograms (RLCs) from cone-beam computed tomography (CBCT) in 85 patients were selected. Computer-assisted manual analysis (Dolphin Imaging 11.9) and AI automatic analysis (Planmeca Romexis 6.2) were used to locate 19 landmarks and obtain 23 measurements. Mean radial error (MRE) and successful detection rate (SDR) values were calculated to assess the accuracy of automatic landmark digitization. Paired *t* tests and Bland‒Altman plots were used to compare the differences and consistencies in cephalometric measurements between manual and automatic analysis programs.

**Results:**

The MRE for 19 cephalometric landmarks was 2.07 ± 1.35 mm with the automatic program. The average SDR within 1 mm, 2 mm, 2.5 mm, 3 and 4 mm were 18.82%, 58.58%, 71.70%, 82.04% and 91.39%, respectively. Soft tissue landmarks (1.54 ± 0.85 mm) had the most consistency, while dental landmarks (2.37 ± 1.55 mm) had the most variation. In total, 15 out of 23 measurements were within the clinically acceptable level of accuracy, 2 mm or 2°. The rates of consistency within the 95% limits of agreement were all above 90% for all measurement parameters.

**Conclusion:**

Automatic analysis software collects cephalometric measurements almost effectively enough to be acceptable in clinical work. Nevertheless, automatic cephalometry is not capable of completely replacing manual tracing. Additional manual supervision and adjustment for automatic programs can increase accuracy and efficiency.

**Supplementary Information:**

The online version contains supplementary material available at 10.1186/s12903-023-02881-8.

## Background

Lateral cephalometry has consistently played an important role in orthodontic diagnosis and treatment protocol design for decades. It can not only clarify the dentofacial morphology and the anatomic basis for malocclusion but also evaluate the growth patterns in the craniofacial complex. Thus, it is significant for orthodontists to locate cephalometric landmarks precisely [1, 2].

Currently, cephalometric analysis is commonly carried out by manual tracing with computer-assisted analysis software [3–5]. Precise definitions of landmarks, calibration of operators and replication of tracings are all important in cephalometric analysis. However, repeat work of landmark localization may spare a great deal of time but do little to improve the accuracy of analysis [6]. The precision, reliability and time demands of cephalometric analysis depend to a great extent on the experience of orthodontists, who must spend a great deal of time training and accumulating experience before they can perform the analysis reliably.

In recent years, a large number of artificial intelligence (AI) studies have been conducted to explore automated landmark locations [7–14]. Compared with traditional cephalometric analysis, AI landmark identification shows superiority in repeatability and efficiency [11, 15]. Although there were several studies comparing the difference of cephalometric analysis between human and AI [16–20], the various automated cephalometric software available to practitioners still need to be evaluated with the development of AI application in orthodontics. Planmeca Romexis software is widely used for cephalometric analysis, and 2-dimensional automatic tracing is feasible and practical with this program. Rather than spending valuable time on training, supporting and monitoring the work of others, clinicians only need a few seconds to obtain the results of cephalometric analysis from it. Therefore, it is important to evaluate the accuracy of this program as a potentially time-saving tool. Our study was designed to compare the automatic localization program with one of the most frequently used computer-assisted cephalometric analysis programs, Dolphin software. Moreover, as far as we know that there have been only a few studies involving both cephalometric landmark and measurement analysis, the accuracy of landmarks and measurements in terms of bone, teeth and soft tissue were evaluated respectively in this study to explore more comprehensively the potential application of AI cephalometric analysis in clinical work.

## Methods

The study was approved by the Ethical Committee Department of the Affiliated Hospital of Stomatology, Nanjing Medical University (PJ2022-030-01).

### Data collection

In this observational study, reconstructed lateral cephalograms (RLCs) processed from cone-beam computed tomography (CBCT) scans were assessed. The retrospective CBCT images obtained from the patient database at the Affiliated Hospital of Stomatology, Nanjing Medical University were collected for the purposes of clinical diagnosis and analysis including airway volume analysis, temporomandibular joint or alveolar bone assessment and so on. A statistical power of 80% at a significance level (alpha) of 0.05 using a two-sided paired *t* test was assumed by the G*power software (version 3.1, Heinrich-Heine-University Dusseldorf, Germany). The calculation showed that 35 images were required with an effect size of 0.49 according to the previous study [17]. For this study, we searched the patient database and selected 85 patients who underwent CBCT from 2015 to 2020.

The sample inclusion criteria were as follows: (1) all CBCT images were taken in a supine position using the same machine and fully included all relevant anatomical structures, and (2) no central incisors were missing or defective. Additionally, the factors of age, gender and skeletal classification were considered during sample inclusion to increase the representativeness of the sample and therefore the applicability of the conclusion. The exclusion criteria were patients who had (1) impacted teeth in the anterior region, (2) prosthetic restoration of the central incisors, (3) previous orthodontic treatment or orthognathic surgery, or (4) cleft lip and palate syndromes.

All CBCT images were obtained with the same CBCT machine (NewTom 5G; Quantitative Radiology, Verona, Italy) with a standard acquisition protocol ( 18 × 16 cm field of view, 110 kV, 1–20 mA pulsed mode, and 0.3-mm voxel size).

### RLC acquisition and processing

CBCT images were reconstructed into RLCs by using Dolphin software (version 11.9, Chatsworth, California, USA). The orthogonal projection type was chosen to create non-distorted images. All RLCs were generated with optimal opacity of hard tissue visualization and adjusted with the slider to sharpen the images. Then, the RLCs were entered into the computer-assisted manual analysis program (Dolphin software) and the automatic cephalometric analysis software (Planmeca Romexis software, version 6.2, Helsinki, Finland).

### Cephalometric analysis

**Fig. 1:**
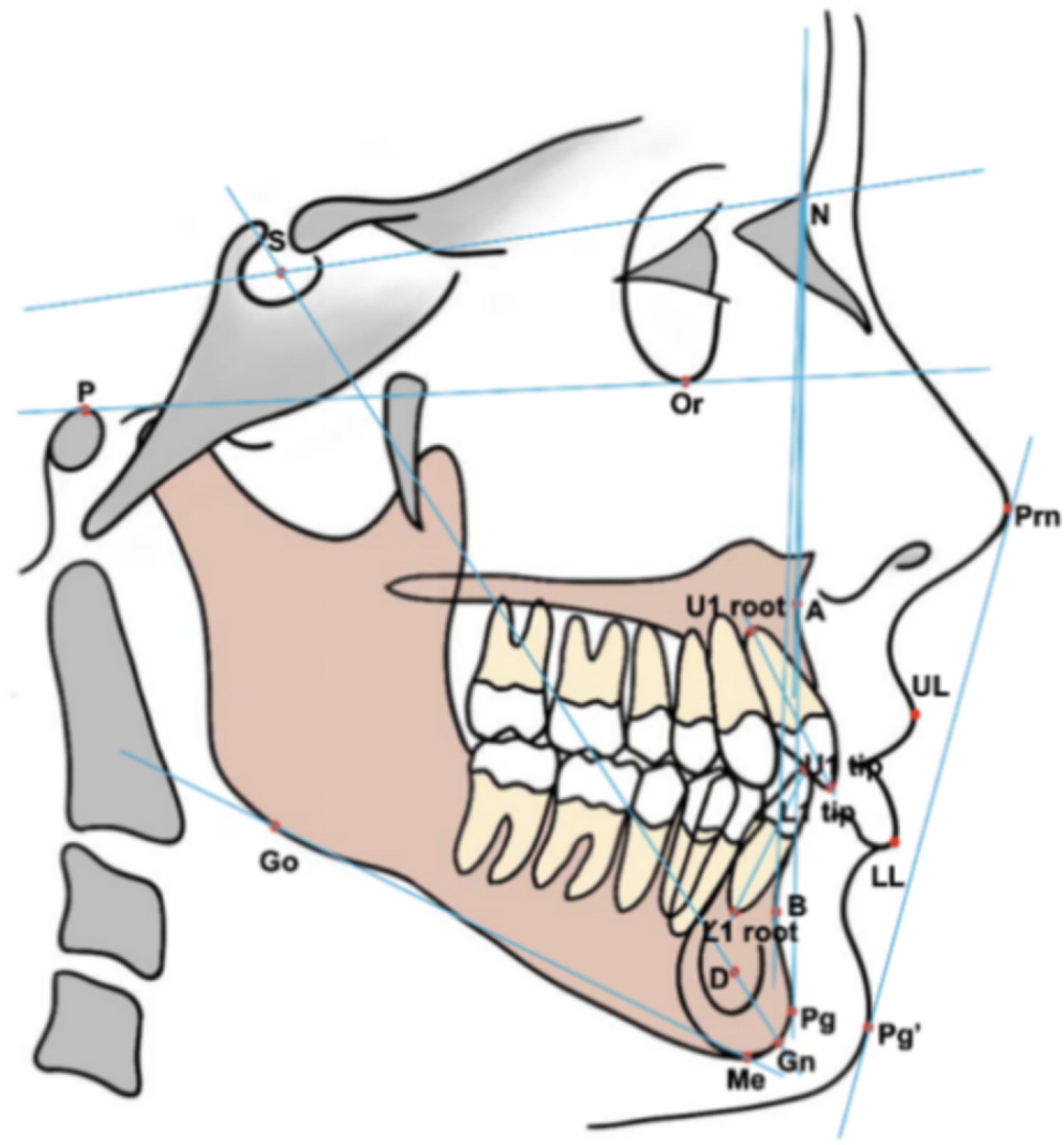
Cephalometric landmarks and measurements in terms of bone, teeth and soft tissue. The definitions of the landmarks and measurements are enumerated in Additional file 1 and Additional file 2

Totally 19 landmarks and 23 measurements (Fig. 1) were chosen on the basis of Steiner, Downs, Tweed cephalometric analysis and some other measurements commonly used in clinical practice. All RLCs were analysed twice by a well-trained orthodontic clinician. The procedure was supervised by two experienced orthodontists (BY and LL, with 17 and 10 years of experience) to ensure the accuracy of manual analysis. No more than 20 images were traced in a single day, and repeat analyses were separated by a two-week interval. The time required for manual tracing was also recorded. Then, the landmark coordinate information and cephalometric measurement values were obtained. To verify the inter-operator precision, another operator (also an orthodontic clinician having undergone the cephalometric training) performed the same process, and the cephalometric measurement values were compared with those of the first operator. The operators identified the landmarks on the same computer.

### Statistical analysis

Analyses were conducted using IBM SPSS, version 23 (IBM Corp., Armonk, NY). Statistical significance was defined as a two-sided *P* value < 0.05. The Shapiro–Wilk test was used to test the normality of the distributions.

The error of landmark positions was identified by superimposing ruler points using the x and y coordinates. The radial error R was calculated between manually detected and AI-detected landmark coordinates:

$$R=\sqrt{{\varDelta x}^{2}+{\varDelta y}^{2}}$$

The mean radial error (MRE), standard deviation (SD) and successful detection rate (SDR) were calculated as follows (in the following equations, N denotes the sample size, and z refers to the precision ranges of 1, 2, 2.5, 3, and 4 mm.)

$$MRE=\frac{\sum _{i=1}^{N}{R}_{i}}{N}$$

$$SD=\sqrt{\sum _{i=1}^{N}{{(R}_{i}-MRE)}^{2}/(N-1)}$$

$$SDR = \frac{\begin{array}{l}{\rm{the}}\,{\rm{number}}\,{\rm{of}}\,{\rm{successfully}}\,{\rm{detected }}\\{\rm{landmarks}}\,{\rm{or}}\,{\rm{measurements}}\,{\rm{within}}\,\\{\rm{the}}\,{\rm{range}}\,{\rm{of}}\,{\rm{z}}\end{array}}{N}{\rm{*100\% }}$$

The MRE; SD; and SDR at 1, 2, 2.5, 3, and 4 mm for 19 landmarks were analysed to evaluate the accuracy of AI-driven localization performance. Moreover, the scatter plots with 95% confidence ellipses were depicted to visualize and evaluate the error pattern in 2-dimensional directions.

The intraclass correlation coefficient (ICC) was analysed to confirm the consistency of the results among multiple measurements. Paired *t* tests; proportion within 95% limits of agreement; and Bland‒Altman plots were used to assess the consistency of manual and AI cephalometric measurements.

**Fig. 2:**
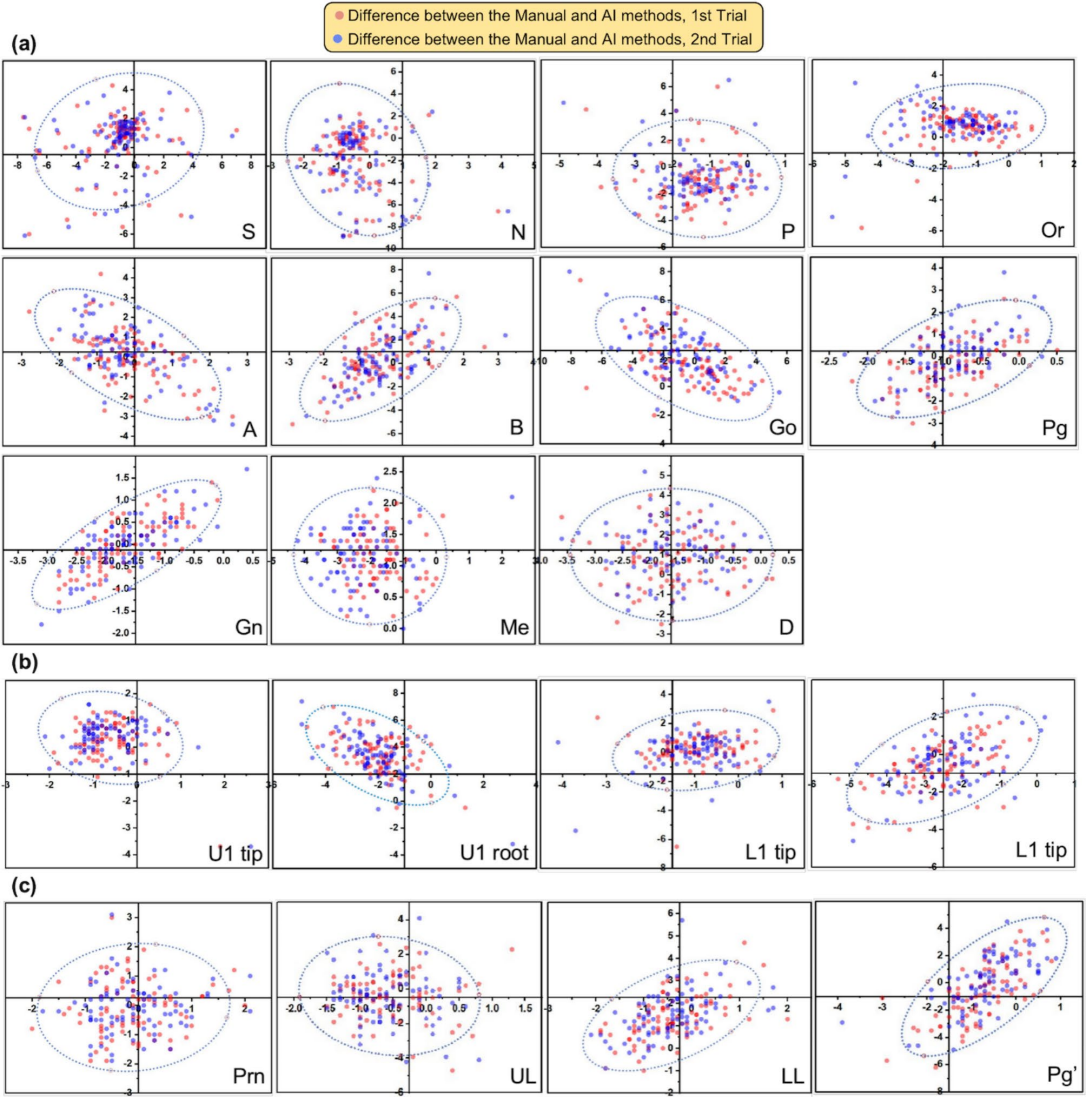
The scatter plots with 95% confidence ellipses show the difference in the coordinates of each landmark between the manual and AI methods. **a** Skeletal landmarks, **b** dental landmarks, **c** soft tissue landmarks. The origin points (0,0) are located by the AI program for different landmarks. The Red and blue points represent the difference between two repetitions of manual digitization and AI detection, respectively

## Results

In this study, we analysed 85 CBCT images, taking into account baseline variables including gender, age, and skeletal classification. The distributions of specific characteristics within the sample are described in Table 1.

**Table 1:** Characteristics of the sample

Variables		N (%)
Total		85 (100%)
Gender	Female	44 (52%)
	Male	41 (48%)
Age	Mixed dentition (11.26 ± 0.79 y)	43 (51%)
	Permanent dentition (19.93 ± 5.82 y)	42 (49%)
Skeletal classification	Class I	39 (46%)
	Class II	15 (18%)
	Class III	31 (36%)

The ICC values showed mean intra-operator reproducibility of 0.97 (0.91–0.99) and 0.96 (0.90–0.99). The ICC of the mean measurement values between the two operators was 0.94 (0.86–0.99) on average. Both the intra-operator and inter-operator comparisons [see Additional file 3] indicated excellent reliability [21]. Given the high consistency between operators, the mean values of operator 1’s analysis were used in this study. The average time required for cephalometry was 157 s for manual tracing and 2 s for AI analysis.

**Table 2:** The bias between manual and automatic detection and the SDR of automatic detection

Landmarks	MRE (mm)	SD (mm)	95% CI (mm)			SDR (%)				
			Lower	Upper	< 1 mm		< 2 mm	< 2.5 mm	< 3 mm	< 4 mm
Skeletal landmarks (11)										
S	2.61	1.88	-1.07	6.29		7.06	57.65	63.53	71.76	81.18
N	2.63	2.31	-1.90	7.16		28.24	55.29	62.35	63.53	77.65
P	2.24	1.14	0.01	4.47		11.76	51.76	62.35	78.82	95.29
Or	1.97	1.07	-0.13	4.07		16.47	58.82	72.94	87.06	96.47
A	1.39	0.87	-0.32	3.10		35.29	77.65	85.88	95.29	98.82
B	1.96	1.19	-0.37	4.29		20.00	61.18	71.76	80.00	94.12
Go	3.08	1.67	-0.19	6.35		3.53	25.88	48.24	58.82	75.29
Pg	1.31	0.57	0.19	2.43		32.94	87.06	96.47	98.82	100.00
Gn	1.77	0.53	0.73	2.81		4.71	65.88	89.41	97.65	100.00
Me	2.41	0.65	1.14	3.68		3.53	20.00	54.12	85.88	98.82
D	2.27	0.86	0.58	3.96		5.88	41.18	64.71	83.53	96.47
Average	2.15	1.37	-0.54	4.84		15.40	54.76	70.16	81.92	92.19
Dental landmarks (4)										
U1 tip	1.06	0.49	0.10	2.02		45.88	97.65	98.82	98.82	98.82
U1 root	4.14	1.33	1.53	6.75		1.18	4.71	9.41	22.35	43.53
L1 tip	1.41	0.86	-0.28	3.10		31.76	87.06	94.12	96.47	97.65
L1 root	2.85	0.93	1.03	4.67		0.00	17.65	35.29	62.35	90.59
Average	2.37	1.55	-0.67	5.41		19.71	51.77	59.41	70.00	82.65
Soft tissue landmarks (4)										
Prn	1.01	0.48	0.07	1.95		51.76	97.65	98.82	98.82	100.00
UL	1.40	0.78	-0.13	2.93		34.12	83.53	91.76	95.29	98.82
LL	1.80	0.74	0.35	3.25		9.41	64.71	85.88	94.12	98.82
Pg′	1.97	0.98	0.05	3.89		14.12	57.65	76.47	89.41	94.12
Average	1.54	0.85	-0.13	3.21		27.35	75.89	88.23	94.41	97.94
Total average	2.07	1.35	-0.58	4.72		18.82	58.58	71.70	82.04	91.39

*SD* Standard deviation; *CI* Confidence interval

### Accuracy in detecting landmarks

The differences in landmark tracing between manual and AI detection and the SDR of automatic detection are shown in Table 2. The detection accuracy of landmarks varied over a large range. The average variation of 19 landmarks was 2.07 ± 1.35 mm. In a comparison of results among the three landmark categories, soft tissue landmarks had the highest consistency (1.54 ± 0.85 mm), while dental landmarks had the lowest consistency (2.37 ± 1.55 mm). Among the 19 landmarks, the Prn point (1.01 ± 0.48 mm) had the highest consistency and the best performance in terms of SDR. Meanwhile, the Pg (1.31 ± 0.57 mm) and U1 tip (1.06 ± 0.49 mm) points almost had the lowest MRE and highest SDR among the skeletal and dental landmarks. The scatter plots (Fig. 2) suggest the distribution of landmark position differences.

**Fig. 3:**
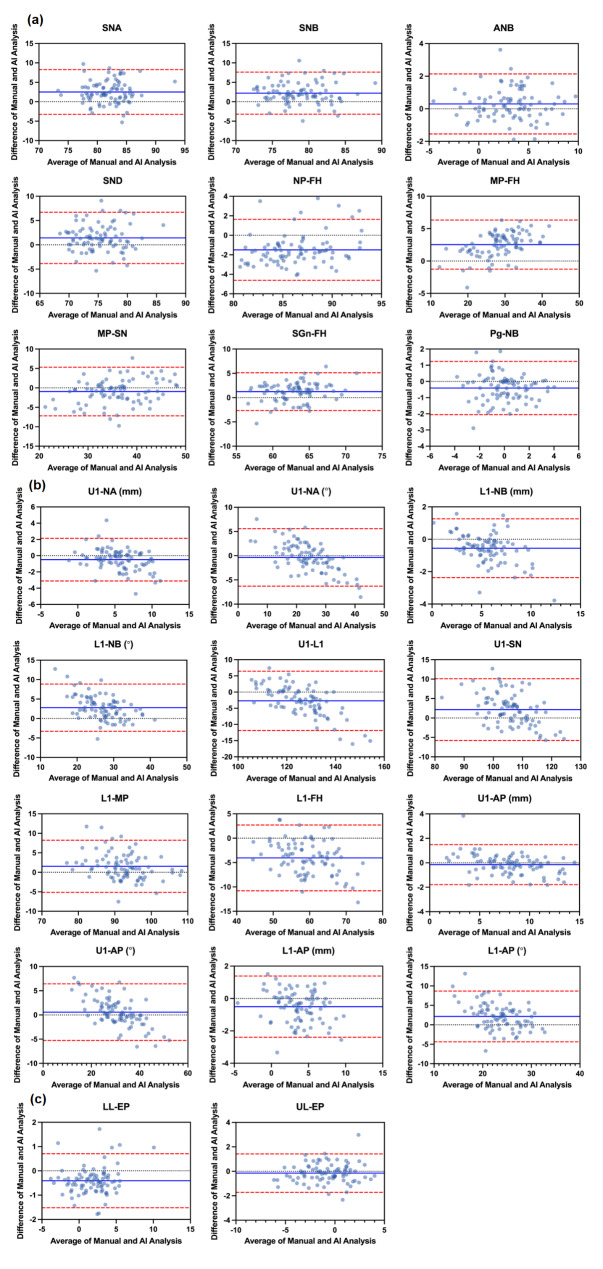
Bland‒Altman plots of cephalometric measurements. **a** Skeletal measurements, **b** dental measurements, **c** soft tissue measurements. For each plot, the x-axis presents the average of manual and AI results, while the y-axis presents the difference between the paired measurements. The blue line represents the mean bias, and the red dashed lines represent the upper and lower 95% limits of agreement. The scale values on the x- and y-axis vary according to the mean and bias of each measurement comparison

### Accuracy in acquiring measurements

Except for 4 cephalometric measurements, U1-NA (°), U1-AP (°), U1-AP (°) and UL-EP (mm), the other difference values of 19 measurements between manual and AI detection were statistically significant (*P <* 0.05) according to a paired *t* test (Table 3). Considering the clinically acceptable limit of 2 mm or 2°, 8 measurements [20], L1-FH (°) (-4.05 ± 3.44), L1-NB (°) (2.78 ± 3.10), U1-L1 (°) (-2.72 ± 4.67), MP-FH (°) (2.53 ± 1.93), SNA (°) (2.50 ± 2.94), SNB (°) (2.21 ± 2.76), L1-AP (°) (2.15 ± 3.33), and U1-SN (°) (2.14 ± 4.06), were discrepant from manual tracing. The Bland‒Altman plots (Fig. 3) represent the consistency of the results between the manual and AI methods. Measurement bias was within the 95% limits of agreement in more than 90% of instances (Table 3).

**Table 3:** Comparison of the cephalometric measurements between manual and AI analyses

Bias	Mean (mm)	SD (mm)	95% limits of agreement (mm)		*P* value^a^	Proportion within 95% limits of agreement (%)
			Lower	Upper		
Skeletal measurements (9)						
SNA (°)	2.50	2.94	-3.25	8.26	0.00	95.29
SNB (°)	2.21	2.76	-3.21	7.62	0.00	95.29
ANB (°)	0.30	0.94	-1.55	2.14	0.01	95.29
SND (°)	1.42	2.69	-3.86	6.69	0.00	94.12
NP-FH (°)	-1.50	1.60	-4.63	1.63	0.00	91.76
MP-FH (°)	2.53	1.93	-1.26	6.32	0.00	96.47
MP-SN (°)	-0.94	3.19	-7.19	5.31	0.01	96.47
Y axis (°)	1.22	1.98	-2.66	5.11	0.00	95.29
Pg-NB (mm)	-0.41	0.84	-2.06	1.23	0.00	95.29
Dental measurements (12)						
U1-NA (mm)	-0.49	1.34	-3.12	2.13	0.00	95.29
U1-NA (°)	-0.36	3.03	-6.29	5.58	0.28	95.29
L1-NB (mm)	-0.56	0.93	-2.37	1.26	0.00	95.29
L1-NB (°)	2.78	3.10	-3.29	8.85	0.00	94.12
U1-L1 (°)	-2.72	4.67	-11.87	6.44	0.00	94.12
U1-SN (°)	2.14	4.06	-5.81	10.09	0.00	98.82
L1-MP (°)	1.52	3.41	-5.17	8.21	0.00	92.94
L1-FH (°)	-4.05	3.44	-10.80	2.70	0.00	95.29
U1-AP (mm)	-0.15	0.84	-1.79	1.48	0.10	96.47
U1-AP (°)	0.57	2.98	-5.28	6.42	0.08	94.12
L1-AP (mm)	-0.51	0.97	-2.40	1.39	0.00	96.47
L1-AP (°)	2.15	3.33	-4.38	8.67	0.00	96.47
Soft tissue measurements (2)						
LL-EP (mm)	-0.41	0.57	-1.52	0.70	0.00	91.76
UL-EP (mm)	-0.15	0.80	-1.72	1.42	0.09	95.29

*SD* Standard deviation

^a^Results of a paired *t* test between manual and AI detection. All data were found to be normally distributed according to the Shapiro‒Wilk test

## Discussion

Cephalometric analysis is essential in every stage of orthodontic treatment. However, manual analysis has low consistency between repetitions and takes a great deal of time. With the rapid development of technology, computer vision allows machines to understand and work with images for cephalometric landmark detection after being trained with a given dataset. In contrast to conventional manual methods, AI can assess images within seconds, helping users save time and increasing the efficiency of repetitive work [11, 15]. According to our study, humans take an average of nearly 80 times as long as the AI program to complete the task. Using an automatic program saves a great deal of time and frees up orthodontists for other tasks. Additionally, the accuracy of cephalometric landmark digitization depends largely on the operator. The results of tracings by different operators or even repeated tracings by the same operator are likely to be significantly different [22]. In contrast to computer-assisted manual tracings, the results obtained from the same automatic program are fairly stable, showing its superiority in terms of repeatability [15]. Nevertheless, whether the accuracy of manual and AI cephalometric analyses is acceptable is always an important concern. To date, growing numbers of studies have focused on the accuracy of the automated cephalometric software, which is more accessible to the practicing orthodontist than in-house algorithms [16–20]. Therefore, our study is practical and meaningful for both the users of automatic programs and the engineers who seek to improve the algorithms.

The results regarding accuracy in landmark detection are consistent with published studies [9, 10]. The most accurately defined points, such as Prn, U1 tip and Pg, are on the edges of anatomical structures with sharp margins on CBCT images. Automatic programs are capable of identifying them with a high degree of precision similar to that of humans. The MREs of the U1 root (4.14 ± 1.33 mm), Go (3.08 ± 1.67 mm), L1 root (2.85 ± 0.93 mm), N (2.63 ± 2.31 mm), S (2.61 ± 1.88 mm), Me (2.41 ± 0.65 mm), D (2.27 ± 0.86 mm) and P points (2.24 ± 1.14 mm) exceeded the average value (2.07 ± 1.35 mm). The great deviation of the U1 root and L1 root points may be due to the indistinct outlines of the incisor roots [10, 23]. The recognition of incisor roots is obstructed by other dental roots when patients have anterior tooth crowding or overbite malocclusion, which is also a challenge for orthodontists in clinical work. The mixed density of incisor roots on imaging makes them difficult to identify by manual or automatic analysis. The reason for the significant errors in the Go, Me and P points is probably the asymmetrical structure of the lower mandible margin and external auditory canal, which varies over a wide range in the population. Midpoints of bilateral structures are chosen when the left and right sides cannot overlap, while asymmetrical structures increase the complexity of the region. Localizing these points relies heavily on the personal judgement of the operator. Obstruction by adjacent anatomical structures may also affect the localization of the Go point, which increases the difficulty of detection. As for the S, N and D points, the relevant structures are sometimes vague and quite ambiguous in RLCs, which makes them difficult for automatic programs to locate. The distance in landmark position between manual localization and automatic localization is interpreted as follows: <2 mm is correct, and < 4 mm is acceptable [24]. In this study, the average SDR as defined by MRE within 1 mm, 2 mm, 2.5 mm, 3 and 4 mm were 18.82%, 58.58%, 71.70%, 82.04% and 91.39%, respectively. Thus, more than half of the points were localized correctly, and more than 90% of the points were localized acceptably well.

The scatter plots (Fig. 2) indicate the distribution of landmark localization error by distance and direction. The 95% confidence ellipse shows a two-dimensional expansion of the Bland-Altman plot to observe the correlation between the x- and y-axis errors in the shape of the ellipse [15]. Points such as Or, UL and LL were distributed along the x-axis, which implied mainly horizontal bias in automatic detection. Similarly, points such as N, B, Gn and Pg’ illustrated mainly vertical bias. The anatomical positions of cephalometric landmarks are subject to bias in both the x and y coordinates. Points with considerable horizontal bias or vertical bias are usually ambiguous to locate in the corresponding direction to a certain extent. The results of AI detection are consistent with those of manual detection.

For cephalometric measurements, the rate of consistency within the 95% limits of agreement was between 91.76% and 98.82% (Table 3), which indicated high consistency in these measurements. Also, the Bland-Altman plots (Fig. 3) visualize the consistency between paired measurements. The results essentially corroborate those of a previous study [17]. The range between the limits of agreement is wider for dental measurements than for skeletal or soft tissue measurements. This conclusion is consistent with our conclusion regarding landmark identification. There were a total of 8 measurements that were both statistically significant (*P*<0.05) and had mean differences above 2 mm or 2°. Dental measurements have more discrepancy in comparison with manual tracing. Soft tissue measurements are basically equivalent in the two methods. Since the RLCs selected in this study were all from people who had not yet undergone orthodontic treatment, the precision of dental measurements might increase if the automatic program were applied to post-treatment images.

Generally, the consistency of AI cephalometric measurements is almost at an acceptable level, considering that it is nearly 80 times faster than manual cephalometric analysis. The main results that were of interest to us proved fairly good precision and consistency with manual analysis. Only a few measurements showed significant bias. Thus, human supervision is still necessary for automatic landmark identification. The solutions for increasing the accuracy and efficiency of automatic cephalometric analysis may be to improve the quality of the image like sharpening and algorithm approving and to implement an outlier detection and feedback system [23]. Orthodontists would need to spend only a short time checking the results of automatic localization, which could save them a great deal of time during clinical work.

Despite its strengths, this study also has several limitations. We used initial diagnosis RLCs to assess the automatic program. Diverse and complex intraoral conditions, such as overlapping teeth, overjet and overbite, are complex and make it difficult for AI to recognize relevant landmarks and obtain measurements. More detailed classification of image samples would increase the comprehensiveness of the conclusions. In addition, we selected fewer soft tissue landmarks than skeletal or dental landmarks, and the same was true of soft tissue measurements versus skeletal and dental measurements. The results would be more reliable if more soft tissue indicators were included.

## Conclusion

Cephalometric measurements obtained from automatic analysis software are almost reliable enough to be acceptable in clinical work. Nevertheless, automatic cephalometry is not capable of completely replacing manual tracing. There still exist differences in localization, especially for some dental landmarks. Additional manual supervision and adjustment of automatic programs will help increase their accuracy, consistency and efficiency.

## Electronic supplementary material

Below is the link to the electronic supplementary material.

Supplementary Material 1

Supplementary Material 2

Supplementary Material 3

## Data Availability

The data analysed in this study are available from the corresponding author on reasonable request.

## Abbreviations

CBCT: Cone beam computer tomography
RLC: Reconstructed lateral cephalogram
MRE: Mean radial error
SDR: Successful detection rate
AI: Artificial intelligence
SD: Standard deviation
ICC: Intraclass correlation coefficient
CI: Confidence interval.
