# Supplementary material for: Evaluating the accuracy of automated cephalometric analysis based on artificial intelligence
Source: BMC Oral Health. 2023 Apr 1;23:191. doi: 10.1186/s12903-023-02881-8 (PMC10067288; doi:10.1186/s12903-023-02881-8)
Supplement: Supplementary file 1 — Supplementary Material 1 [file 12903_2023_2881_MOESM1_ESM.docx]

**Additional file 1**

**Table S1.** Definition of cephalometric landmarks used in this study

| Landmarks (abbreviation) | Definition |
| --- | --- |
| Skeletal landmarks (11) | |
| Sella (S) | The midpoint of the cavity of sella turcica.. |
| Nasion (N) | The anterior point of the intersection between the nasal and frontal bones. |
| Porion (P) | The midpoint of the upper contour of the external auditory canal. |
| Orbitale (Or) | The lowest point on the inferior margin of the orbit. |
| Point A (A) | The innermost point on the contour of the premaxilla between anterior nasal spine and the incisor tooth. |
| Point B (B) | The innermost point on the contour of the mandible between the incisor tooth and the bony chin. |
| Gonion (Go) | The most posterior and inferior point at the angle of the mandible. |
| Pogonion (Pg) | The most anterior point on the contour of the chin. |
| Gnathion (Gn) | The most anterior and inferior point on the contour of the chin. |
| Menton (Me) | The most inferior point on the contour of the chin. |
| Center of symphysis (D) | The center point of the mandibular symphysis. |
| Dental landmarks (4) | |
| U1 tip | The point of tip of upper central incisor. |
| U1 root | The point of root of upper central incisor. |
| L1 tip | The point of tip of lower central incisor. |
| L1 root | The point of root of lower central incisor. |
| Soft tissue landmarks (4) | |
| Pronasale (Prn) | The most prominent point of apex nasi. |
| Upper lip (UL) | The most prominent point of the border of the upper lip. |
| Lower lip (LL) | The most prominent point of the border of the lower lip. |
| Soft tissue pogonion (Pg’) | The most anterior soft tissue point of the chin in the midsagittal plane. |
