# Supplementary material for: Evaluating the accuracy of automated cephalometric analysis based on artificial intelligence
Source: BMC Oral Health. 2023 Apr 1;23:191. doi: 10.1186/s12903-023-02881-8 (PMC10067288; doi:10.1186/s12903-023-02881-8)
Supplement: Supplementary file 2 — Supplementary Material 2 [file 12903_2023_2881_MOESM2_ESM.docx]

**Additional file 2**

**Table S2.** Definition of cephalometric measurements used in this study

| Measurements | Definition |
| --- | --- |
| Skeletal measurements (9) | |
| SNA (°) | The angle formed between points S, N, and A. |
| SNB (°) | The angle formed between points S, N, and B. |
| ANB (°) | The angle formed between points A, N, and B. |
| SND (°) | The angle formed between points S, N, and D. |
| NP-FH (°) | The angle formed by line N-Pg and Frankfort horizontal plane (P-Or). |
| MP-FH (°) | The angle formed by mandibular plane (Go-Me) and Frankfort horizontal plane (P-Or). |
| MP-SN (°) | The angle formed by mandibular plane (Go-Me) and line S-N. |
| Y axis (°) | The angle formed by line S-Gn and Frankfort horizontal plane (P-Or). |
| Pg-NB (mm) | The distance between point Pg and line N-B. |
| Dental measurements (12) | |
| U1-NA (mm) | The distance between point U1 tip and line N-A. |
| U1-NA (°) | The angle formed by upper incisor axes and line N-A. |
| L1-NB (mm) | The distance between point L1 tip and line N-B. |
| L1-NB (°) | The angle formed by lower incisor axes and line N-B. |
| U1-L1 (°) | The angle formed by the intersection of upper incisor and lower incisor axes. |
| U1-SN (°) | The angle formed by upper incisor axes and line S-N. |
| L1-MP (°) | The angle formed by lower incisor axes and mandibular plane (Go-Me). |
| L1-FH (°) | The angle formed by lower incisor axes and Frankfort horizontal plane (P-Or). |
| U1-AP (mm) | The distance between point U1 tip and line A-Pg. |
| U1-AP (°) | The angle formed by upper incisor axes and line A-Pg. |
| L1-AP (mm) | The distance between point L1 tip and line A-Pg. |
| L1-AP (°) | The angle formed by lower incisor axes and line A-Pg. |
| Soft tissue measurements (2) | |
| LL-EP (mm) | The distance between point LL and E plane (line Prn-Pg’). |
| UL-EP (mm) | The distance between point UL and E plane (line Prn-Pg’). |
