# Supplementary material for: Evaluating the accuracy of automated cephalometric analysis based on artificial intelligence
Source: BMC Oral Health. 2023 Apr 1;23:191. doi: 10.1186/s12903-023-02881-8 (PMC10067288; doi:10.1186/s12903-023-02881-8)
Supplement: Supplementary file 3 — Supplementary Material 3 [file 12903_2023_2881_MOESM3_ESM.docx]

**Additional file 3**

**Table S3.** ICC values of intra-operator and inter-operator analysis

| Reliability | | | |
| --- | --- | --- | --- |
|  | intra-operator 1 | intra-operator 2 | inter-operator |
| Skeletal measurements (9) | | | |
| SNA (°) | 0.99 | 0.94 | 0.87 |
| SNB (°) | 0.99 | 0.97 | 0.93 |
| ANB (°) | 0.99 | 0.98 | 0.97 |
| SND (°) | 0.99 | 0.96 | 0.93 |
| NP-FH (°) | 0.97 | 0.96 | 0.97 |
| MP-FH (°) | 0.98 | 0.98 | 0.94 |
| MP-SN (°) | 0.99 | 0.98 | 0.96 |
| Y axis (°) | 0.96 | 0.95 | 0.96 |
| Pg-NB (mm) | 0.96 | 0.96 | 0.91 |
| Dental measurements (12) | | | |
| U1-NA (mm) | 0.98 | 0.96 | 0.96 |
| U1-NA (°) | 0.98 | 0.96 | 0.91 |
| L1-NB (mm) | 0.97 | 0.99 | 0.97 |
| L1-NB (°) | 0.94 | 0.94 | 0.95 |
| U1-L1 (°) | 0.98 | 0.97 | 0.92 |
| U1-SN (°) | 0.98 | 0.95 | 0.86 |
| L1-MP (°) | 0.95 | 0.95 | 0.95 |
| L1-FH (°) | 0.95 | 0.95 | 0.95 |
| U1-AP (mm) | 0.99 | 0.99 | 0.99 |
| U1-AP (°) | 0.98 | 0.97 | 0.91 |
| L1-AP (mm) | 0.98 | 0.98 | 0.99 |
| L1-AP (°) | 0.91 | 0.90 | 0.91 |
| Soft tissue measurements (2) | | | |
| LL-EP (mm) | 0.99 | 0.98 | 0.99 |
| UL-EP (mm) | 0.98 | 0.99 | 0.93 |
